# Total ionizing dose (TID) effects of γ ray radiation on switching behaviors of Ag/AlO_*x*_/Pt RRAM device

**DOI:** 10.1186/1556-276X-9-452

**Published:** 2014-08-29

**Authors:** Fang Yuan, Zhigang Zhang, Jer-Chyi Wang, Liyang Pan, Jun Xu, Chao-Sung Lai

**Affiliations:** 1 Institute of Microelectronics, Tsinghua University, Tsinghua Yuan 1st, Haidian District, Beijing 100084, China; 2 Department of Electronic Engineering, Chang Gung University, 259 Wen-Hwa 1st Road, Kwei-Shan 333 Tao-Yuan, Taiwan

**Keywords:** Total ionizing dose (TID) effects, *γ* ray radiation, RRAM, Hybrid filament model, Filament, Radiation-induced holes

## Abstract

The total ionizing dose (TID) effects of ^60^Co *γ* ray radiation on the resistive random access memory (RRAM) devices with the structure of Ag/AlO_*x*_/Pt were studied. The resistance in low resistance state (LRS), set voltage, and reset voltage are almost immune to radiation, whereas the initial resistance, resistance at high resistance state (HRS), and forming voltage were significantly impacted after radiation due to the radiation-induced holes. A novel hybrid filament model is proposed to explain the radiation effects, presuming that holes are co-operated with Ag ions to build filaments. In addition, the thermal coefficients of the resistivity in LRS can support this hybrid filament model. The Ag/AlO_*x*_/Pt RRAM devices exhibit radiation immunity to a TID up to 1 Mrad(Si) and are highly suitable for radiation-hard electronics applications.

## Background

Recently, resistive random access memory (RRAM) has drawn great research attention. It is widely recognized to be a promising nonvolatile memory for the next generation due to its high compatibility with complementary metal-oxide-semiconductor (CMOS) process and outstanding memory performance such as fast switching speed, high storage density, low power consumption, great data reliability, etc [1-7]. In addition, the future application of RRAM in aerospace or nuclear industry is full of potential. The major challenges in such applications lie in the radiation-induced degradation of RRAM performance. Radiation sources in the outer aerospace and nuclear industries include X-ray and *γ* ray radiation, energetic electrons, protons, and heavy ion bombardment, etc., and they can bring displacement damages, radiation-induced charge trapping on oxide layers, radiation-induced tunneling leakage, soft breakdown, and hard breakdown [8-10]. Some studies have pointed out that a few kinds of RRAM materials have a good immunity to certain types of radiation, such as HfO_2_[11,12], TiO_2_[13,14], and Ta_2_O_5_[15,16], etc. The reported good radiation immunity can be ascribed to the reversible filament-based switching mechanism of these RRAM devices. When an operation voltage is applied to the RRAM device, metal ions or oxygen ions/vacancies from the device electrodes or from the oxide material, according to the electrical field, drift in the film bulk to form or rupture the conducting filaments, leading the device transit between the high and low resistance states reversibly [17-20]. Similarly, aluminum oxide (AlO_*x*_), which is widely used in modern CMOS technology, also has an excellent filament-based RRAM performance [2,3]. However, the radiation effects on AlO_*x*_ RRAM are not implemented.

In this work, the filament-based RRAM with the structure of Ag/AlO_*x*_/Pt was chosen as the experimental devices since it has the well-understood filament-based switching mechanism. ^60^Co *γ* ray treatment is used as the radiation source to investigate the total ionizing dose (TID) effects on the devices. The switching behaviors and memory performances with different radiation doses are compared and analyzed. Moreover, a radiation-induced hybrid filament model is proposed to explain the TID effects of *γ* ray treatment.

## Methods

**Figure 1:**
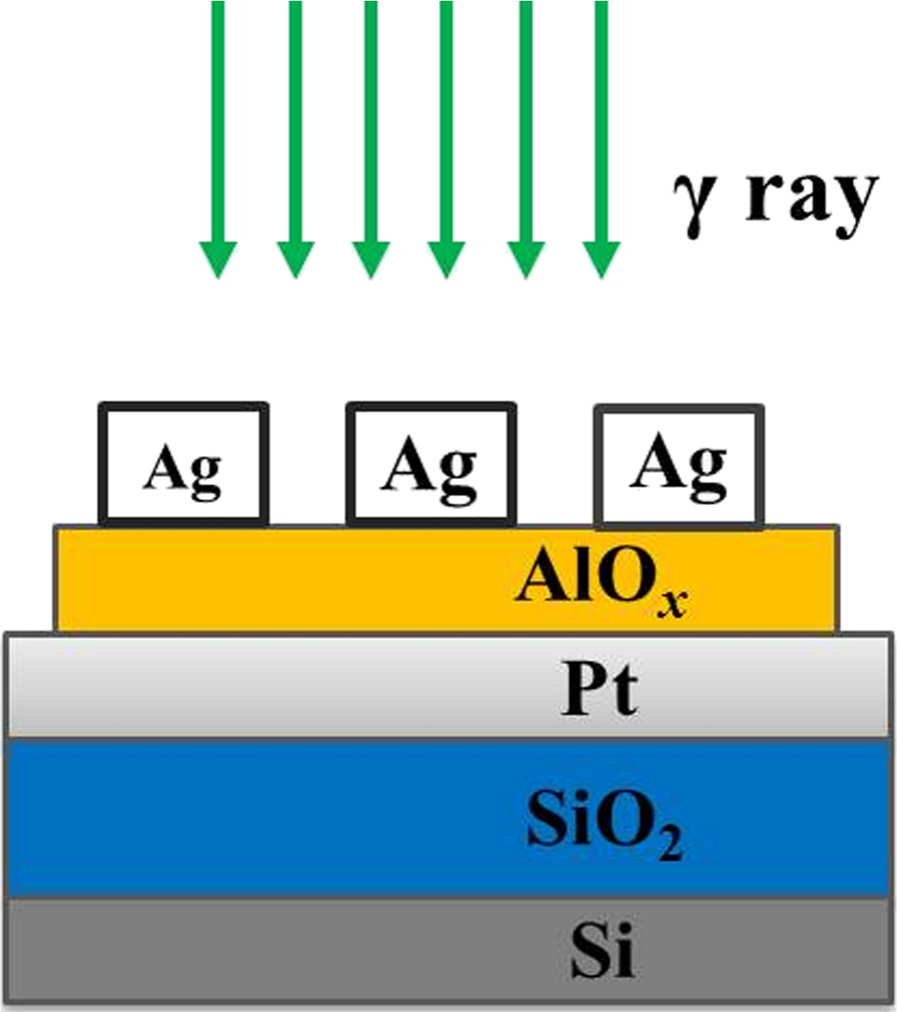
**Schematic illustration of the Ag/AlO**_***x***_**/Pt RRAM devices.** The ^60^Co *γ* ray radiation is performed after the device is fabricated.

Ag/AlO_*x*_/Pt RRAM devices were fabricated for the radiation study. After a standard Radio Corporation of America (RCA) cleaning of the p-type silicon wafers, a 300-nm-thick silicon dioxide was thermally grown as an isolation layer. Then a 100-nm-thick Pt film was deposited by the e-beam evaporator as a bottom electrode (BE). Next, a 20-nm-thick AlO_*x*_ film, as resistive switching layer, was deposited by the atomic layer deposition (ALD) at 220°C by using the precursors of trimethylaluminium (TMA) and H_2_O. After that, a 100-nm-thick Ag film was deposited and patterned by the shadow mask method to form the top electrode (TE). The schematic diagram of the Ag/AlO_*x*_/Pt RRAM devices is shown in Figure 1. After the RRAM devices have been fabricated, some of the as-made devices were exposed to ^60^Co *γ* ray radiation at the rate of 100 rad(Si)/s with the total dose up to 500 krad(Si) and 1 Mrad(Si). The electrical characteristics of the RRAM devices were measured using an Agilent 1500A precise semiconductor analyzer (Agilent Technologies; Santa Clara, CA, USA) on a variable temperature probe station. The bias was applied at TE, and the BE was connected to ground.

## Results and discussion

**Figure 2:**
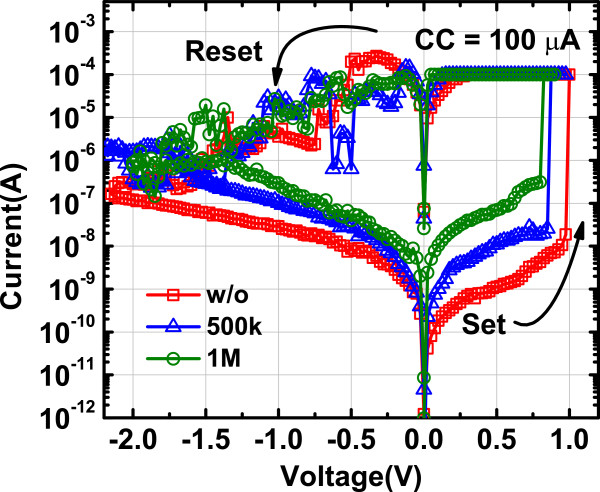
**Typical *I-V *curves of Ag/AlO**_***x***_**/Pt RRAM devices with different total radiation dose.** The bipolar resistive switching is still stable after the *γ* ray radiation.

Figure 2 shows the typical current versus voltage (*I-V*) curves of the Ag/AlO_*x*_/Pt RRAM devices with different radiation total dose. A forming process is needed to firstly turn the devices on. All samples exhibit stable bipolar switching behaviors with set and reset voltages at approximately +1.0 and -2.0 V, respectively, so that the switching mode has not been changed by the radiation. The switching mechanism of this kind of RRAM devices has been well studied, which is the formation and rupture of the metallic filaments (Ag) in the oxide film at positive and negative TE bias, respectively [17-20].

**Figure 3:**
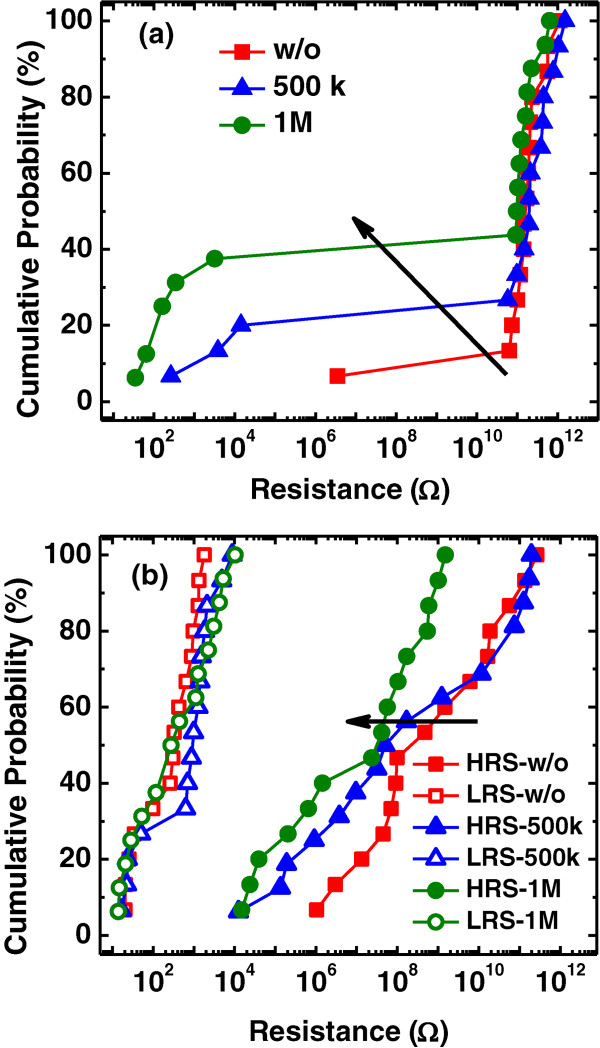
**Resistance distributions of the Ag/AlO**_***x***_**/Pt RRAM devices.** Distribution of **(a)** the initial resistance and **(b)** the resistance in HRS and LRS of the devices with different radiation doses. A degeneration of the initial resistance and the resistance in HRS occurs for the radiated samples.

To investigate the TID radiation impact on the performance of resistive switching memory, at least 15 samples of each RRAM device were measured and analyzed by using a statistical method. Figure 3a shows the initial resistance of the devices, in which an obvious degeneration of uniformity can be found. The resistance reduction of some samples can be observed after the radiation, and the amount of low-resistance samples increases with the radiation dose. It is resulted from the radiation-induced soft breakdown in AlO_*x*_ film of the RRAM device, and several conducting paths are created by the radiation [21]. As the radiation dose increases, there arise more conducting channels within the film, turning more fresh devices to the low resistance. The initial resistance failure can be recovered by a reset operation through a negative TE bias sweep, bringing the device back to the high resistance state (HRS). Figure 3b presents the distribution of the resistance in HRS and low resistance state (LRS) for the samples. It is reported that holes will be generated by the *γ* ray in AlO_*x*_ film, and an increase of tunneling leakage current can be induced by these holes [22]. The resistance at HRS is mainly determined by the resistance of the resistive switching layer [11], so that the increase of leakage paths will lead to the decrease of resistance at HRS. On the other hand, the resistance in LRS is mostly related to the Ag filament. Thus, there is nearly no change of the resistance in LRS after the *γ* ray radiation.

**Figure 4:**
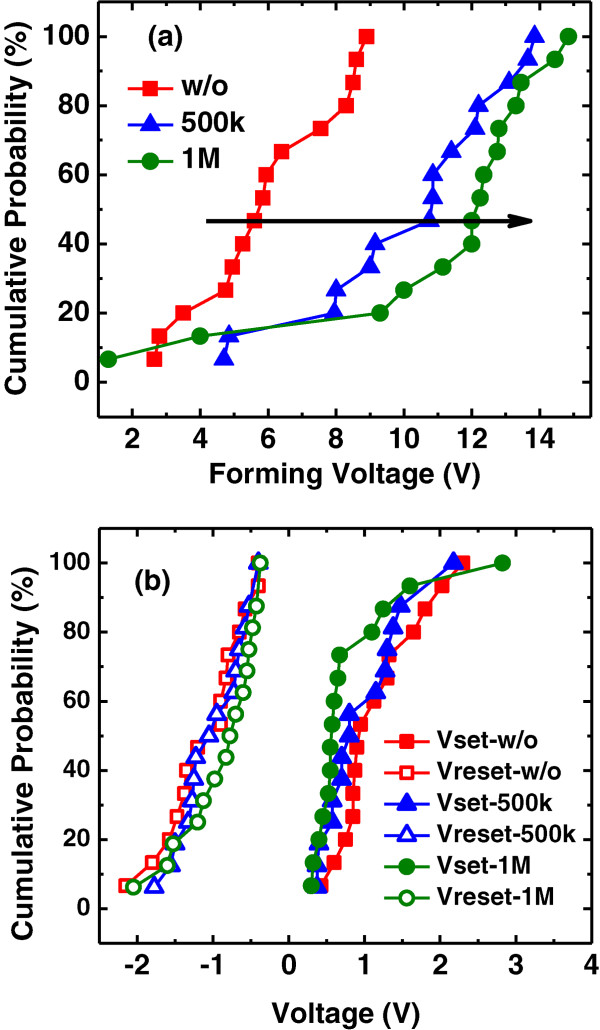
**Operation voltage distributions of the Ag/AlO**_***x***_**/Pt RRAM devices.** Distribution of **(a)** the forming voltage and **(b)** the set and reset voltages with different doses of radiation. An obvious increase in forming voltage and a slight decrease in set voltage are observed.

The distributions of forming voltages and set and reset voltages are demonstrated in Figure 4a and b, respectively. A severe increase to over +10 V of forming voltage is observed for the samples with *γ* ray radiation, whereas a slight change of set and reset voltages can be observed. For the forming process, the scattering of Ag ions is reinforced by the *γ* ray radiation and more Ag ions have migrated into the film bulk [11]. Simultaneously, radiation arouses defects and trapped charges inside the film which needs a stronger electrical field to fulfill or recombine. Therefore, a higher forming voltage is needed to realize the first filament gathering and penetration. It is noticeable that the first operation to set the device to LRS is defined as forming process, also for the devices with a low initial resistance and recovered by a reset operation. As for the set process, the radiation-induced holes assist the formation of the Ag filament and result in a slight decrease of set voltage. While for the reset process, the filament rupture is related to the drift of Ag ions under the reset voltage-induced electrical field, therefore the role of the radiation-induced holes can be ignored [11]. Although the radiation leads to a scattering of Ag ions into the film bulk, this scattering influence on the set and reset procedures is almost negligible. After forming operations, several filaments have been built inside the film bulk, and during the following set and reset operations, the rupture and the reconnection of the filaments only occurs within a relatively local region, near the electrode interface.

**Figure 5:**
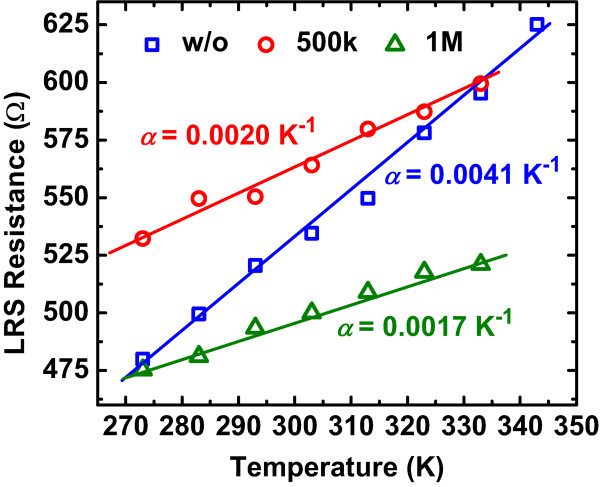
**Temperature dependence of resistance in LRS.** The symbols are experiment data, and the lines are fitting results. The values of *α* indicate a change of the metal-like characteristics in filaments as the radiation dose increases.

**Figure 6:**
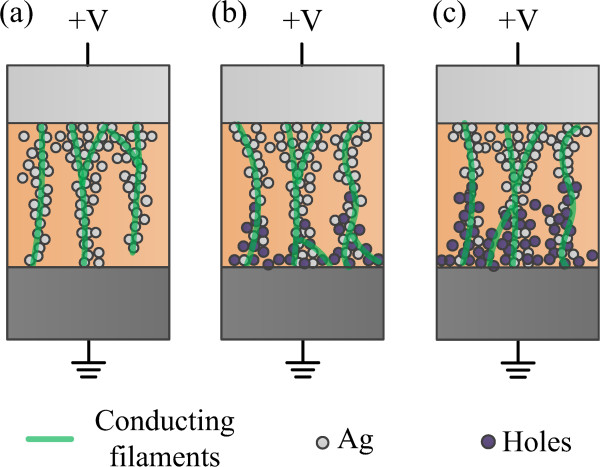
**Schematic diagrams of the proposed hybrid filament model for the radiation effects.** The schematic diagram of filaments in LRS of the devices **(a)** without radiation, and with the total radiation dose of **(b)** 500 krad(Si) and **(c)** 1 Mrad(Si). The microscopic changes of the filaments reveal an increase of holes generated by the radiation.

As the discussion described above, the effects of holes generated by the *γ* ray radiation are important for the resistive switching of Ag/AlO_*x*_/Pt RRAM devices. In order to clarify the role of the radiation-induced holes, an elevated temperature measurement was carried out. The temperature dependence of resistance in LRS of the samples is studied, and the thermal coefficients of resistivity (*α*) are calculated and shown in Figure 5. The *α* value of the devices without radiation is extracted to be 0.0041 K^-1^, which is quite close to the proposed value of 0.0038 K^-1^ for the high-purity silver at 293 K [23], meaning that the major constituent of conducting filaments in LRS is silver. Interestingly, the *α* values become smaller as the radiations dose increases, which are 0.0020 and 0.0017 K^-1^ for the device of 500 krad(Si) and 1 Mrad(Si) dose, respectively. The increase implies that the metal-like characteristic of the filaments changes as the radiation dose increases. The change is due to the holes aroused by *γ* ray radiation, which co-form the conducting filaments of Ag ions in LRS. Therefore, a hybrid filament model is developed to illustrate the change of RRAM devices after radiation. When the device is exposed to *γ* ray radiation, electron–hole pairs are generated. Some of the electron-hole pairs recombine, while others drift or hop due to the built-in electric field which is caused by the work function difference between the Ag TE and the Pt BE. During the drift or hopping process, most holes are trapped near the BE interface [15,22]. Figure 6 illustrates the low resistance state (conducting filaments have formed and connected two electrodes) of the devices with different radiation doses. A larger radiation dose brings more holes at the bottom interface. In the set process, when a positive voltage is applied to the TE, Ag ions from TE move towards the BE to form the conducting filament. For the devices with *γ* ray radiation, the induced holes participate in the growth of filaments and, that is, narrow the distance for Ag ions to drift. Furthermore, the holes create more parallel filaments near the BE interface and a little decrease of set voltage and the resistance in LRS can be observed, as shown in Figures 3b and 4b. As for the reset process, a negative voltage attracts Ag ions back to TE, which is not affected by the holes, so that a little change has been found between these samples. Thus, the constituent of filaments in LRS becomes hybrid after *γ* ray radiation, which is proved by the thermal coefficients extracted from the resistivity in LRS as shown in Figure 5.

Table 1 lists a comparison of the radiation effects between three reported RRAM materials and this work. From the comparison, the RRAM device in this work exhibits a satisfied immunity to high dose *γ* ray radiation. The degeneration tendency of LRS resistance, HRS resistance, and operation voltages after radiation almost agree with the literature. While the decrease of initial resistance is opposite to the reported result in [15], which is possibly due to the different oxygen-vacancy-governed switching mechanism of TiN/TaO_*x*_/Pt devices.

**Table 1:** Comparison of radiation effects between published literature and this work

**References**	**Device structure**	**Total dose**	**Resistance**			**Operation voltage**		
			**Initial**	**Low**	**High**	**Forming**	**Set**	**Reset**
[11]	Cu/HfO_2_:Cu/Pt	360 krad (Si)	NA	√	↓	√	↑	√
[13]	Pt/TiO_2_/Pt	14 Mrad (Si)	NA	√	√	NA	NA	NA
[15]	TiN/TaO_*x*_/Pt	180 krad (Si)	↑	√	↓	NA	NA	NA
**This work**	Ag/AlO_*x*_/Pt	1 Mrad (Si)	↓	√	↓	↑	√	√

NA, not available; √, no or negligible change; ↑, increase; ↓, decrease.

## Conclusions

In this paper, the total ionizing dose (TID) effect of ^60^Co *γ* ray radiation on Ag/AlO_*x*_/Pt RRAM devices has been investigated. Degradations of uniformity and performance are observed in resistance and switching voltage, which is caused by the radiation-induced holes. A hybrid filament model is proposed to suggest that holes are co-operated with Ag ions to build filaments. The model is proved by the thermal coefficients of resistivity in LRS. Moreover, the Ag/AlO_*x*_/Pt RRAM devices demonstrate a satisfactory anti-radiation ability because of the stable resistive switching and a sufficient memory window.

## Competing interests

The authors declare that they have no competing interests.

## Authors’ contributions

FY and ZZ provide the idea and designed this study. FY performed the experiments under the guidance of JX and LP. J-CW and C-SL participated in the coordination of the study. All authors discussed the results. FY completed the manuscript. All authors read and approved the final manuscript.
